# Network analysis uncovers putative genes affecting resistance to tick infestation in Braford cattle skin

**DOI:** 10.1186/s12864-019-6360-3

**Published:** 2019-12-19

**Authors:** Daniela D. Moré, Fernando F. Cardoso, Maurício A. Mudadu, Wilson Malagó-Jr, Claudia C. Gulias-Gomes, Bruna P. Sollero, Adriana M. G. Ibelli, Luiz L. Coutinho, Luciana C. A. Regitano

**Affiliations:** 1 EMBRAPA Pecuária Sudeste, São Carlos, São Paulo, Brazil; 2 EMBRAPA Pecuária Sul, Bagé, Rio Grande do Sul Brazil; 3 Federal University of Pelotas, Capão do Leão, Rio Grande do Sul Brazil; 4 EMBRAPA Informática Agropecuária, Campinas, São Paulo, Brazil; 5 EMBRAPA Suínos e Aves, Concórdia, Santa Catarina Brazil; 6 Luiz de Queiroz College of Agriculture, University of São Paulo, Piracicaba, São Paulo, Brazil

**Keywords:** *Rhipicephalus microplus*, Bovine, Braford, Gene expression, RNA-Seq, Enrichment analysis

## Abstract

**Background:**

Genetic resistance in cattle is considered a suitable way to control tick burden and its consequent losses for livestock production. Exploring tick-resistant (R) and tick-susceptible (S) hosts, we investigated the genetic mechanisms underlying the variation of Braford resistance to tick infestation. Skin biopsies from four-times-artificially infested R (*n* = 20) and S (*n* = 19) hosts, obtained before the first and 24 h after the fourth tick infestation were submitted to RNA-Sequencing. Differential gene expression, functional enrichment, and network analysis were performed to identify genetic pathways and transcription factors (TFs) affecting host resistance.

**Results:**

Intergroup comparisons of hosts before (R*pre* vs. S*pre*) and after (R*post* vs. S*post*) tick infestation found 51 differentially expressed genes (DEGs), of which almost all presented high variation (TopDEGs), and 38 were redundant genes. Gene expression was consistently different between R and S hosts, suggesting the existence of specific anti-tick mechanisms. In the intragroup comparisons, R*post* vs. *Rpre* and S*post* vs. S*pre*, we found more than two thousand DEGs in response to tick infestation in both resistance groups. Redundant and non-redundant TopDEGs with potential anti-tick functions suggested a role in the development of different levels of resistance within the same breed. Leukocyte chemotaxis was over-represented in both hosts, whereas skin degradation and remodeling were only found in TopDEGs from R hosts. Also, these genes indicated the participation of cytokines, such as IL6 and IL22, and the activation of Wingless (WNT)-signaling pathway. A central gene of this pathway, *WNT7A,* was consistently modulated when hosts were compared. Moreover, the findings based on a genome-wide association study (GWAS) corroborate the prediction of the WNT-signaling pathway as a candidate mechanism of resistance. The regulation of immune response was the most relevant pathway predicted for S hosts. Members of Ap1 and NF-kB families were the most relevant TFs predicted for R and S, respectively.

**Conclusion:**

This work provides indications of genetic mechanisms presented by Braford cattle with different levels of resistance in response to tick infestation, contributing to the search of candidate genes for tick resistance in bovine.

## Background

Cattle are the preferential hosts of *Rhipicephalus microplus*, a hard tick that attaches to the host skin and feeds for three weeks. Tick attachment and feeding depend upon numerous saliva components that inhibit host hemostatic responses to the parasite bites [1], a process that is the result of millions of years of evolution [2]. *Rhipicephalus microplus* is the most important ectoparasite of livestock, especially in tropical and subtropical areas [3], causing severe illness in cattle [4], with annual global costs of around US$ 22–30 billion [5].

Acaricides are currently the most common tick control method. However, significant levels of resistance to the different acaricide classes [6, 7] along with potential contamination of milk, beef, and the environment no longer support their use. Vaccination is an alternative for tick control, and several efforts have been conducted to increase its effectiveness [8–10]. Genetic resistance can be a permanent solution to tick control [11]. Bovine resistance to *R. microplus* infestation is a heritable phenotype, and heritability values around 0.34 were observed across different populations [12, 13]. Tick resistance has been studied in several cattle breeds [14–18], and *Bos taurus taurus* breeds are more susceptible to tick infestation compared to *Bos taurus indicus* breeds [11]. Braford, a composite breed of 3/8 Zebu (*B. t. indicus*) and 5/8 Hereford (*B. t. taurus*), presents considerable variation in tick resistance.

Previous attempts to understand the genetic mechanisms underlying resistance explored host immune responsiveness. However, differences in experimental design hamper comparison of results [19, 20]. Tick response was primarily compared between zebuine and taurine cattle breeds [14, 21–27], but differences have been reported between low and high resistance levels within the same breed [28–31]. Overall, these studies show an essential role of the structural protein-coding genes and cellular immunity through innate and acquired mechanisms, including cytokines, chemokines, T cells, B cells, mast cells, and granulocytes. Although some cellular characterization has been done, bovine gene expression has been mainly assessed by RT-PCR and microarray assays, limiting differential gene expression analyses in terms of the number of genes investigated. Studies from Australia [32] and Brazil [33–36] have identified Quantitative Trait Loci (QTL) underlying host variation. While the Australian study tested a candidate gene (integrin alpha 11), Brazilian studies were either based on microsatellite technology, resulting in wide confidence intervals for QTL locations, or on TagSNPs. Though these studies brought to light some aspects of genetic influence in tick resistance, they did not investigate, in a more comprehensive manner, which genes and pathways are involved in resistance. Jonsson et al., 2014 [14], reviewing genetic marker research, suggested that cell-mediated immunity, hypersensitivity, local inflammation and structural skin components contribute to host resistance. Porto-Neto et al. [37] validated a positional candidate gene (receptor-interacting serine-threonine kinase 2, *RIPK2*) for tick burden using a knock-out mice model. Genomic approaches to explore genetic variation affecting tick host resistance have been reviewed [38].

In this report we used high throughput RNA sequencing technology to compare gene expression in tick resistant and susceptible Braford cattle. Functional enrichment and network analyses were employed to uncover genetic mechanisms of host resistance to tick infestation. The mechanisms identified could contribute to the understanding of host immunity against ticks.

## Results

### Differential gene expression analysis

**Fig. 1:**
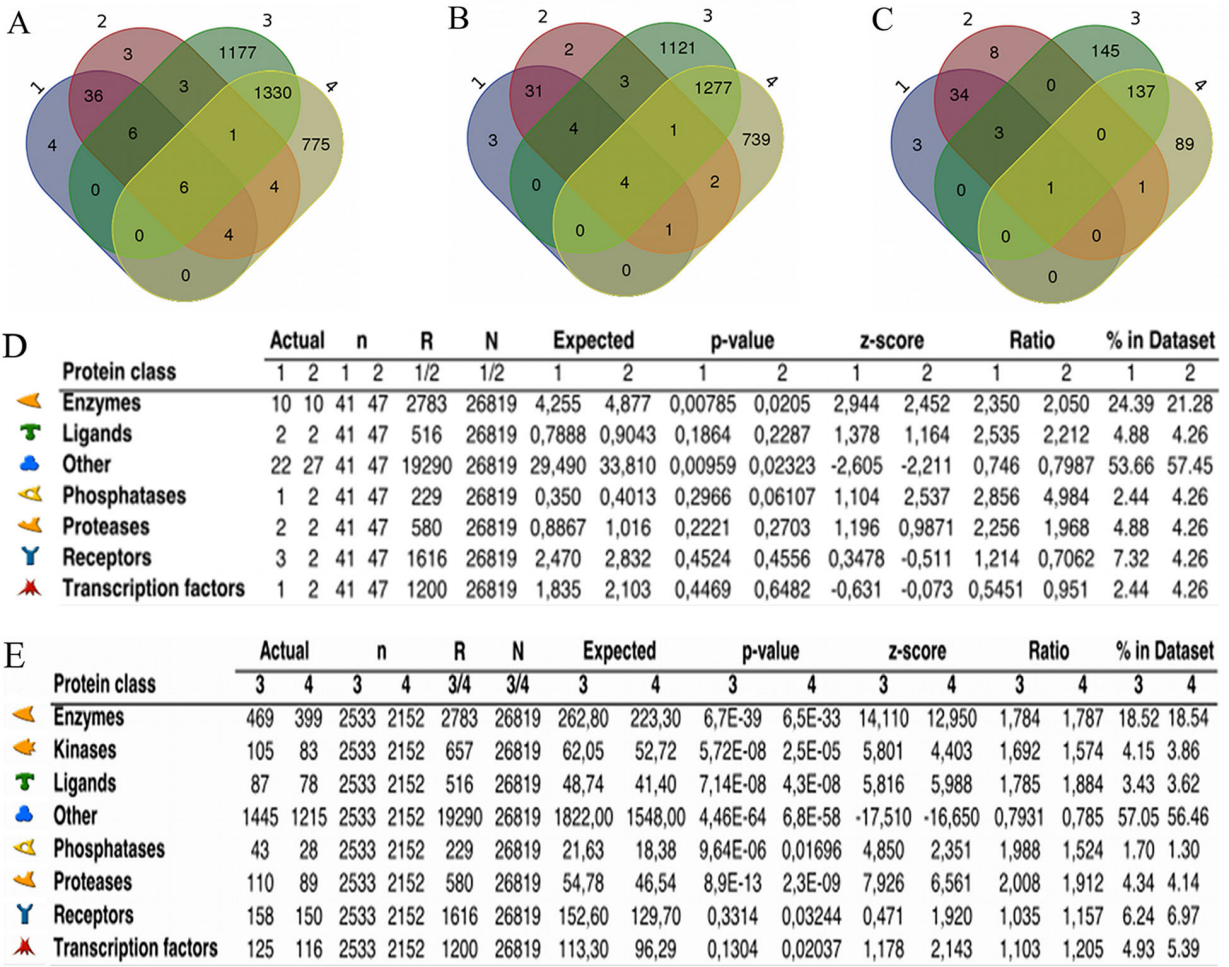
Functional enrichment of differentially expressed genes (DEGs) in Braford skin according to host phenotype and tick infestation. Venn diagrams show the distribution of DEGs from inter- (R*pre* vs. S*pre* (1) and R*post* vs. S*post* (2)) and intragroup (R*post* vs. R*pre* (3) and S*post* vs. S*pre* (4)) comparisons: (**a**) all DEGs, (**b**) only the annotated ones, or (**c**) only annotated TopDEGs. Functional annotation based on protein classes of DEGs from (**d**) inter- and (**e**) intragroup comparisons are shown, represented by a symbol following the Metacore® reference guide (https://portal.genego.com/legends/MetaCoreQuickReferenceGuide.pdf). Actual: number of network objects from the dataset(s) for a given protein class; n: number of network objects in the dataset(s); R: number of network objects of a given protein class in the complete database or background list; N: total number of network objects in the complete database or background list; Expected: mean value for hypergeometric distribution (n*R/N); *p*-value: probability to have the given value of Actual or higher (or lower for negative z-score); z-score: ((Actual-Expected)/sqrt(variance)); Ratio: connectivity ratio (Actual/Expected); % in Dataset: fraction of network objects with a selected function in the dataset

A comprehensive study of skin transcriptomic profile of genetically divergent hosts regarding anti-tick resistance was performed using RNA-Seq technology. Data from 20 resistant (R) and 19 susceptible (S) animals, previously selected from an original population of 974 animals, were collected prior to first artificial tick infestation (*pre*) and 24 h after the fourth infestation (*post*). This strategy was employed to address both innate and acquired immunity before the challenge and those elicited by it. The number of reads after filtering was around 10 M per sample. No differences among groups were observed for the mapping statistics with the reads presenting around 70% concomitant pair alignment rate at the gene level. Read information and mapping statistics are presented in Additional file 1: Table S1. Fig. 1 shows Venn diagrams illustrating the distribution of differentially expressed genes (DEGs) from intergroup: R*pre* vs. S*pre* (1) and R*post* vs. S*post* (2) and intragroup: R*post* vs. R*pre* (3) and S*post* vs. S*pre* (4) comparisons, including unknown genes (Fig. 1a), only the annotated genes (Fig. 1b), and only the annotated TopDEGs (|log_2_| FC > 1) (Fig. 1c).

At a false discovery rate (FDR) < 0.05, R*pre* vs. S*pre* (1) showed 56 DEGs, with 35 up- and 21 down-regulated (Table 1 and Fig. 1a), and 43 annotated genes (Table 1 and Fig. 1b). R*post* vs. S*post* (2) showed 63 DEGs, with 37 up- and 26 down-regulated (Table 1 and Fig. 1a), and 48 annotated genes (Table 1 and Fig. 1b). Among annotated DEGs, 41 genes are TopDEGs in R*pre* vs. *Spre* and 47 in R*post* vs. *Spost* (Fig. 1c), with overall fold change ranging from 7.59 to − 9.59 (Table 1). The intergroup comparisons showed 38 redundant TopDEGs (Fig. 1c, intersection between 1 and 2). The correlation (*r*^2^) among log_2_ FC values from these redundant TopDEGs was 0.99.

**Table 1:** Intergroup comparisons of differentially expressed genes in the skin of tick-resistant and -susceptible Braford cattle

Gene Symbol	Ensembl Gene ID	Description	log_2_ FC^a^	FDR^b^
*ALOX12E*	ENSBTAG00000031933	*Bos taurus* arachidonate lipoxygenase, epidermal, mRNA. [Source:RefSeq mRNA;Acc:NM_001083532]	3.17	7.10E-07^1^
			3.58	1.41E-08^2^
*ANXA8*	ENSBTAG00000018499	*Bos taurus* annexin A8-like 1 (ANXA8L1), mRNA. [Source:RefSeq mRNA;Acc:NM_174241]	1.28	5.14E-04^1^
			1.34	2.16E-04^2^
*ASIP*	ENSBTAG00000034077	*Bos taurus* agouti signaling protein, mRNA. [Source:RefSeq mRNA;Acc:NM_206843]	4.56	3.74E-08^1^
			4.74	6.12E-09^2^
*AWAT1*	ENSBTAG00000018839	*Bos taurus* acyl-CoA wax alcohol acyltransferase 1, mRNA. [Source:RefSeq mRNA;Acc:NM_001192683]	−9.60	1.37E-05^1^
			−9.35	2.73E-05^2^
*BOLA*	ENSBTAG00000022590	uncharacterized protein [Source:UniProtKB/TrEMBL;Acc:F1MWX3]	−2.06	1.17E-03^1^
			2.20	3.93E-04^2^
*CCS*	ENSBTAG00000004343	*Bos taurus* copper chaperone for superoxide dismutase, mRNA. [Source:RefSeq mRNA;Acc:NM_001046187]	1.09	2.29E-02^1^
			1.18	5.96E-03^2^
*COL11A1*	ENSBTAG00000021217	*Bos taurus* collagen, type XI, alpha 1, mRNA. [Source:RefSeq mRNA;Acc:NM_001166509]	−2.04	2.41E-02^1^
			−2.00	2.26E-02^2^
*CPVL*	ENSBTAG00000007146	carboxypeptidase, vitellogenic-like [Source:HGNC Symbol;Acc:14399]	−3.53	2.23E-10^1^
			−3.28	7.47E-09^2^
*CYP2B6*	ENSBTAG00000003871	cytochrome P450, family 2, subfamily B, polypeptide 6 [Source:HGNC Symbol;Acc:2615]	−6.49	1.23E-15^1^
			−6.61	3.02E-16^2^
*DGCR6L*	ENSBTAG00000047299	*Bos taurus* DiGeorge syndrome critical region gene 6 (DGCR6), mRNA. [Source:RefSeq mRNA;Acc:NM_001110446]	0.96	2.12E-02^1^
			1.00	9.24E-03^2^
*DHRS4*	ENSBTAG00000017665	*Bos taurus* dehydrogenase/reductase (SDR family) member 4, mRNA. [Source:RefSeq mRNA;Acc:NM_174822]	2.03	9.35E-14^1^
			2.11	5.04E-15^2^
*DPYSL4*	ENSBTAG00000017780	*Bos taurus* dihydropyrimidinase-like 4, mRNA. [Source:RefSeq mRNA;Acc:NM_001163783]	6.14	1.73E-02^1^
*ENPP3*	ENSBTAG00000020196	*Bos taurus* ectonucleotide pyrophosphatase/phosphodiesterase 3, mRNA. [Source:RefSeq mRNA;Acc:NM_001075923]	2.65	3.29E-05^1^
			2.69	2.39E-05^2^
*FAM174A*	ENSBTAG00000045909	Bos *taurus* family with sequence similarity 174, member A, mRNA. [Source:RefSeq mRNA;Acc:NM_001206184]	1.29	3.09E-02^1^
			1.47	3.50E-03^2^
*FAM229B*	ENSBTAG00000033429	*Bos taurus* protein FAM229B, mRNA. [Source:RefSeq mRNA;Acc:NM_001195067]	−2.67	3.34E-04^1^
			−2.70	2.29E-04^2^
*FNBP1L*	ENSBTAG00000004383	formin binding protein 1-like [Source:HGNC Symbol;Acc:20851]	−0.81	1.73E-02^1^
			−0.78	2.87E-02^2^
*FOS*	ENSBTAG00000004322	*Bos taurus* FBJ murine osteosarcoma viral oncogene homolog, mRNA. [Source:RefSeq mRNA;Acc:NM_182786]	3.32	6.34E-03^1^
			3.30	9.79E-03^2^
*FUT5*	ENSBTAG00000000414	*Bos taurus* fucosyltransferase 5 (alpha (1,3) fucosyltransferase), mRNA. [Source:RefSeq mRNA;Acc:NM_176851]	2.43	1.10E-02^1^
			2.69	1.79E-03^2^
*GAL3ST1*	ENSBTAG00000014232	*Bos taurus* galactose-3-O-sulfotransferase 1, mRNA. [Source:RefSeq mRNA;Acc:NM_001101972]	1.93	1.60E-02^2^
*GSTM1*	ENSBTAG00000031788	*Bos taurus* glutathione S-transferase mu 1, mRNA. [Source:RefSeq mRNA;Acc:NM_001083772]	2.35	3.29E-05^1^
			2.45	1.28E-05^2^
*HBG2*	ENSBTAG00000037644	*Bos taurus* hemoglobin, gamma 2 (LOC788610), mRNA. [Source:RefSeq mRNA;Acc:NM_001110509]	−4.64	1.99E-03^1^
			− 4.06	2.51E-02^2^
*IMP3*	ENSBTAG00000012199	*Bos taurus* IMP3, U3 small nucleolar ribonucleoprotein, homolog (yeast), mRNA. [Source:RefSeq mRNA;Acc:NM_001079588]	1.50	3.34E-09^1^
			1.39	9.66E-08^2^
*KIF23*	ENSBTAG00000009983	*Bos taurus* kinesin family member 23, mRNA. [Source:RefSeq mRNA;Acc:NM_001098038]	−1.51	1.10E-02^1^
			−1.39	2.72E-02^2^
*LAMA1*	ENSBTAG00000018160	laminin, alpha 1 [Source:HGNC Symbol;Acc:6481]	2.59	4.34E-03^1^
			3.18	9.21E-05^2^
*LOC781726*	ENSBTAG00000038366	LOC781726 protein; Uncharacterized protein [Source:UniProtKB/TrEMBL;Acc:A8YXZ3]	7.31	9.21E-06^1^
			7.16	2.39E-05^2^
*MGC140681*	ENSBTAG00000011692	*Bos taurus* chromosome 25 open reading frame, human C16orf45 (C25H16orf45), mRNA. [Source:RefSeq mRNA;Acc:NM_001078077]	3.71	2.13E-04^1^
			3.29	2.39E-03^2^
*MOSC2*	ENSBTAG00000016277	*Bos taurus* MOCO sulphurase C-terminal domain containing 2, mRNA. [Source:RefSeq mRNA;Acc:NM_001076380]	−1.53	3.13E-03^1^
			−1.52	3.23E-03^2^
*MSRB3*	ENSBTAG00000044017	methionine sulfoxide reductase B3 [Source:HGNC Symbol;Acc:27375]	1.33	1.79E-03^1^
			1.31	2.14E-03^2^
*MTHFS*	ENSBTAG00000020023	*Bos taurus* 5,10-methenyltetrahydrofolate synthetase (5-formyltetrahydrofolate cyclo-ligase), mRNA. [Source:RefSeq mRNA;Acc:NM_001075616]	−1.17	1.41E-02^1^
*MYADML*	ENSBTAG00000002786	*Bos taurus* myeloid-associated differentiation marker-like (LOC781494), mRNA. [Source:RefSeq mRNA;Acc:NM_001101279]	−2.40	1.46E-02^2^
	ENSBTAG00000034302	*Bos taurus* myeloid-associated differentiation marker-like (LOC512150), mRNA. [Source:RefSeq mRNA;Acc:NM_001104975]	6.33	1.67E-03^2^
	ENSBTAG00000040580	*Bos taurus* myeloid-associated differentiation marker-like (LOC618633), mRNA. [Source:RefSeq mRNA;Acc:NM_001103302]	−5.88	3.24E-02^2^
*NDUFC2*	ENSBTAG00000018188	*Bos taurus* NADH dehydrogenase (ubiquinone) 1, subcomplex unknown, 2, 14.5 kDa, mRNA. [Source:RefSeq mRNA;Acc:NM_176642]	1.14	7.06E-03^1^
			1.13	7.84E-03^2^
*NENF*	ENSBTAG00000000759	*Bos taurus* neudesin neurotrophic factor, mRNA. [Source:RefSeq mRNA;Acc:NM_001076419]	1.12	1.13E-02_1_
			1.06	2.32E-02^2^
*PON3*	ENSBTAG00000034645	*Bos taurus* paraoxonase 3, mRNA. [Source:RefSeq mRNA;Acc:NM_001075479]	−4.50	1.95E-17^1^
			−4.43	4.40E-17^2^
*PRODH*	ENSBTAG00000047676	*Bos taurus* proline dehydrogenase (oxidase) 1, mRNA. [Source:RefSeq mRNA;Acc:NM_001075185]	1.51	2.56E-02^1^
			1.52	2.21E-02^2^
*RBP1*	ENSBTAG00000020028	*Bos taurus* retinol binding protein 1, cellular, mRNA. [Source:RefSeq mRNA;Acc:NM_001025343]	1.29	2.54E-02^2^
*RESP18*	ENSBTAG00000010897	*Bos taurus* regulated endocrine-specific protein 18 homolog (rat), mRNA. [Source:RefSeq mRNA;Acc:NM_001077897]	6.70	7.27E-05^1^
			6.57	5.44E-04^2^
*SAA3*	ENSBTAG00000022396	*Bos taurus* serum amyloid A 3, mRNA. [Source:RefSeq mRNA;Acc:NM_181016]	5.05	3.24E-02^1^
			5.55	1.28E-02^2^
*SAO*	ENSBTAG00000001041	*Bos taurus* amine oxidase, copper containing 3, mRNA. [Source:RefSeq mRNA;Acc:NM_001130764]	6.46	4.22E-03^1^
			5.76	1.68E-02^2^
*SIRT5*	ENSBTAG00000014904	*Bos taurus* sirtuin 5, mRNA. [Source:RefSeq mRNA;Acc:NM_001034295]	1.14	1.18E-03^1^
			1.16	8.01E-04^2^
*SLC6A16*	ENSBTAG00000030543	solute carrier family 6, member 16 [Source:HGNC Symbol;Acc:13622]	4.50	6.55E-03^1^
			4.60	3.83E-03^2^
*SYT5*	ENSBTAG00000002522	*Bos taurus* synaptotagmin V, mRNA. [Source:RefSeq mRNA;Acc:NM_001083744]	1.85	4.83E-03^1^
			1.67	2.26E-02^2^
*TACR2*	ENSBTAG00000021664	*Bos taurus* tachykinin receptor 2, mRNA. [Source:RefSeq mRNA;Acc:NM_174469]	2.34	8.25E-03^1^
			2.39	5.10E-03^2^
*TET1*	ENSBTAG00000037756	tet methylcytosine dioxygenase 1 [Source:HGNC Symbol;Acc:29484]	−2.16	4.12E-02^2^
*TNMD*	ENSBTAG00000021059	*Bos taurus* tenomodulin, mRNA. [Source:RefSeq mRNA;Acc:NM_001099948]	−3.93	3.08E-02^1^
			−4.08	1.68E-02^2^
*TNNT3*	ENSBTAG00000022158	*Bos taurus* troponin T type 3 (skeletal, fast), mRNA. [Source:RefSeq mRNA;Acc:NM_001001441]	−3.77	1.68E-02^2^
*TTC36*	ENSBTAG00000014899	*Bos taurus* tetratricopeptide repeat domain 36, mRNA. [Source:RefSeq mRNA;Acc:NM_001040515]	−2.94	1.07E-02^1^
			−2.70	2.72E-02^2^
*UPK3A*	ENSBTAG00000009913	*Bos taurus* uroplakin 3A, mRNA. [Source:RefSeq mRNA;Acc:NM_174709]	3.11	2.26E-02^2^
*UTS2R*	ENSBTAG00000018170	urotensin 2 receptor [Source:HGNC Symbol;Acc:4468]	3.29	5.99E-03^1^
*WNT7A*	ENSBTAG00000001668	*Bos taurus* wingless-type MMTV integration site family, member 7A, mRNA. [Source:RefSeq mRNA;Acc:NM_001192788]	7.10	3.67E-02^1^
			7.59	1.58E-02^2^

^a^log_2_ Fold Change

^b^False Discovery Rate

^1^Resistant vs. Susceptible prior to infestation: R*pre* vs. S*pre*

^2^Resistant vs. Susceptible post infestation: R*post* vs. S*post*

Regarding cellular metabolism, many TopDEGs were implicated in cell activation against injury. We observed functions such as cellular signaling, ion-dependent vesicular trafficking and transport, free radical depuration, and detoxification of products of oxidative stress with chaperone for superoxide dismutase (*CCS)* and glutathione S-transferase mu 1 (*GSTM1*) genes; cytoskeleton organization with kinesin family member 23 (*KIF23*); ribosomal processing with U3 small nucleolar ribonucleoprotein (*IMP3*); cellular growth and differentiation with wingless-type MMTV integration site family member 7A (*WNT7A*) and neudesin neurotrophic factor (*NENF*); as well as regulation of gene expression with FBJ murine osteosarcoma viral oncogene homolog (*FOS*) (Table 1). Genes are listed alphabetically with their Ensembl identification and description.

Concerning response against ticks and immunity, TopDEGs found in both comparisons such as epidermal arachidonate lipoxygenase (*ALOX12E*), acyl-CoA wax alcohol acyltransferase 1 (*AWAT1*), serum amyloid A 3 (*SAA3*) and tachykinin receptor 2 (*TACR2*), have products acting on inflammation. For instance, *AWAT1* showed the greatest modulation among all DEGs, being down-regulated in R*pre* compared to S*pre* and in R*post* compared to S*post* (respectively log_2_ FC − 9.59 and − 9.35), whereas *SSA3* (log_2_ FC 5.05 and 5.55), *TACR2 (*log_2_ FC 2.34 and 2.39) and *WNT7A* (log_2_ FC 7.10 and 7.59) genes were strongly up-regulated in the same comparisons. Unique TopDEGs as dihydropyrimidinase-like 4 (*DPYSL4*) and urotensin 2 receptor (*UTS2R*) were up-regulated only in R*pre* with high log_2_ FC (6.14 and 3.29, respectively), whereas 5,10-methenyltetrahydrofolate synthetase (*MTHFS*) was down-regulated (log_2_ FC − 1.17). TopDEGs such as galactose-3-O-sulfotransferase 1 (*GAL3ST1*), retinol binding protein 1 (*RBP1*), tetmethylcytosinedioxygenase 1 (*TET1*), troponin T type 3 (*TNNT3*), uroplakin 3A (*UPK3A*) and four incompletely annotated myeloid-associated differentiation marker-like (*MYADML*) genes were modulated only in R*post* vs. S*post* (Table 1).

In the intragroup comparisons, R hosts presented 2523 (FDR < 0.05) DEGs after tick infestation, of these 1807 were up- and 716 were down-regulated (Fig. 1a and Additional file 2: Table S2), with 95.52% (2410) annotated (Fig. 1b and Additional file 2: Table S2). The log_2_ FC varied from 5.28 to − 3.77, and about 12.5% (316) were TopDEGs with 200 up- and 116 down-regulated genes. Of these, 286 DEGs were annotated (Fig. 1c and Additional file 2: Table S2), with a considerable number of uncharacterized or non-annotated genes remaining among TopDEGs. Susceptible hosts showed 2120 significant (FDR < 0.05) DEGs after tick infestation, with 1442 up- and 678 down-regulated genes (Fig. 1a and Additional file 2: Table S2), from which 2024 (95.47%) were annotated (Fig. 1b and Additional file 2: Table S2). The log_2_ FC variation was similar to that observed for R, from 5.23 to − 3.65 with 260 TopDEGs (228 annotated), 146 (130) up- and 114 (98) down-regulated genes (Fig. 1c and Additional file 2: Table S2). TopDEGs were distributed over the whole host genome in both phenotypes (except for chromosome 20 in S hosts). We also found unique and redundant DEGs (and TopDEGs) in the intragroup comparisons (Fig. 1). Almost two thousand genes were modulated exclusively in one resistance-group (1177 DEGs, 1121 annotated for R and 775 DEGs, 739 annotated, for S). Nonetheless, more than a thousand DEGs (1337, 1282 annotated) were redundant for both resistance groups after tick infestation with a highly correlated variation on expression (*r*^2^ = 0.97). These genes point to mechanisms elicited by tick challenge independent of the host phenotype, such as immune response, coagulation, vascularization, and ion transport.

### Enrichment analysis (EA) based on functional ontologies

Functional enrichment based on non-redundant TopDEGs predicted more than 30 gene functions, with several categories activated after infestation (*p*-values ranging from 5.84E-03 to 1.55E-07 and z-score > 2), and only one inhibited (p-value 3.43E-04 and z-score < − 2) in R hosts (Table 2). Unique TopDEGs were classified according to the functional category and specific function they represent. Most of the activated functions are somehow related to anti-tick response, as cellular activation and migration, inflammation, lipid metabolism, molecule transport and blood vessel formation. Activation and chemotaxis were observed for all immune cell types such as leukocytes, phagocytes, and granulocytes, and mostly represented by cytokines, chemokines, growth factors, and inflammatory genes in R hosts (Table 2).

**Table 2:** Functional enrichment of non-redundant differentially expressed genes presenting higher variation in the skin of tick-resistant Braford cattle after tick infestation

Category	Function Annotation	*p*-value	z-score^a^	Genes^b^
Behavior	behavior	3.43E-04	−2.24	*CRHR2*, *CSF2*, *DDO*, *DMRTA1*, *DRD3*, *GAD2*, *GAL*, *GRIA2*, *GRIK2*, *GRP*, *HBA1*/*HBA2*, *IL6*, *MAP6*, *NPTX2*, *SLC26A4*, *SYN1*
Cardiovascular System Development and Function	development of cardiovascular system	4.14E-04	2.19	*C6*, *CCR3*, *CRHR2*, *CSF2*, *FOSL1*, *HBA1*/*HBA2*, *HP*, *IL13RA2*, *IL20*, *IL24*, *IL6*, *MMP13*, *OLR1*, *OPTC*, *RETN*, *TNNT2*, *WNT7A*
Cardiovascular System Development and Function, Organismal Development	angiogenesis	3.14E-04	2.19	*C6*, *CCR3*, *CRHR2*, *CSF2*, *FOSL1*, *HP*, *IL13RA2*, *IL20*, *IL24*, *IL6*, *MMP13*, *OLR1*, *OPTC*, *RETN*, *WNT7A*
	vasculogenesis	1.37E-03	2.18	*C6*, *CCR3*, *CRHR2*, *CSF2*, *IL13RA2*, *IL20*, *IL24*, *IL6*, *MMP13*, *OLR1*, *OPTC*, *RETN*
Cell-To-Cell Signaling and Interaction	activation of cells	2.53E-04	3.38	*C6*, *CD79B*, *CSF2*, *DRD3*, *F2RL3*, *GAD2*, *GRIK2*, *IL22*, *IL24*, *IL37*, *IL6*, *LBP*, *MMP13*, *RETN*, *SOST*, *TREML2*
Cell-To-Cell Signaling and Interaction, Cellular Growth and Proliferation	stimulation of cells	1.34E-03	2.04	*CCL20*, *CSF2*, *GAL*, *GRP*, *IL22*, *IL24*, *IL6*
Cell-To-Cell Signaling and Interaction, Cellular Movement, Hematological System Development and Function, Immune Cell Trafficking	recruitment of granulocytes	1.55E-07	2.13	*CCL20*, *CCR3*, *COCH*, *CSF2*, *GRP*, *IL22*, *IL37*, *IL6*, *OLR1*, *TREML2*
Cell-To-Cell Signaling and Interaction, Cellular Movement, Hematological System Development and Function, Immune Cell Trafficking, Inflammatory Response	recruitment of neutrophils	3.89E-07	2.21	*CCL20*, *COCH*, *CSF2*, *GRP*, *IL22*, *IL37*, *IL6*, *OLR1*, *TREML2*
Cell-To-Cell Signaling and Interaction, Hematological System Development and Function	activation of blood cells	1.39E-04	2.95	*C6*, *CD79B*, *CSF2*, *DRD3*, *F2RL3*, *GAD2*, *GRIK2*, *IL22*, *IL24*, *IL37*, *IL6*, *LBP*, *RETN*, *TREML2*
Cell-To-Cell Signaling and Interaction, Hematological System Development and Function, Immune Cell Trafficking, Inflammatory Response	activation of leukocytes	2.93E-03	3.09	*C6*, *CD79B*, *CSF2*, *DRD3*, *GAD2*, *IL22*, *IL24*, *IL37*, *IL6*, *LBP*, *TREML2*
	activation of phagocytes	2.26E-04	2.92	*C6*, *CSF2*, *DRD3*, *IL22*, *IL24*, *IL37*, *IL6*, *LBP*, *TREML2*
	activation of myeloid cells	1.20E-04	2.90	*C6*, *CSF2*, *DRD3*, *IL22*, *IL24*, *IL37*, *IL6*, *LBP*, *TREML2*
	activation of mononuclear leukocytes	1.92E-03	2.55	*CD79B*, *CSF2*, *DRD3*, *GAD2*, *IL22*, *IL24*, *IL6*, *LBP*, *TREML2*
	activation of macrophages	1.61E-03	2.39	*C6*, *CSF2*, *DRD3*, *IL37*, *IL6*, *LBP*
Cellular Movement	migration of cells	7.32E-06	2.74	*C6*, *CCL20*, *CCR3*, *COCH*, *CSF2*, *DRD3*, *F2RL3*, *FAT3*, *FGFR4*, *FOSL1*, *GRIA2*, *GRP*, *HP*, *IGFBP1*, *IL20*, *IL22*, *IL24*, *IL37*, *IL6*, *LBP*, *MGAT3*, *MMP13*, *NPTX2*, *OLR1*, *RETN*, *TREML2*, *VIL1*, *WNT7A*
	cell movement	8.23E-06	2.51	*C6*, *CCL20*, *CCR3*, *COCH*, *CSF2*, *DRD3*, *F2RL3*, *FAT3*, *FGFR4*, *FOSL1*, *GRIA2*, *GRP*, *HP*, *IGFBP1*, *IL13RA2*, *IL20*, *IL22*, *IL24*, *IL37*, *IL6*, *LBP*, *LRRC6*, *MGAT3*, *MMP13*, *NPTX2*, *OLR1*, *RETN*, *TREML2*, *VIL1*, *WNT7A*
	chemotaxis of cells	2.32E-04	2.17	*CCL20*, *CCR3*, *CSF2*, *DRD3*, *GRP*, *HP*, *IL20*, *IL22*, *IL6*, *LBP*, *TREML2*
	chemotaxis	7.34E-05	2.17	*CCL20*, *CCR3*, *CSF2*, *DRD3*, *FOSL1*, *GRP*, *HP*, *IL20*, *IL22*, *IL6*, *LBP*, *TREML2*
Cellular Movement, Hematological System Development and Function, Immune Cell Trafficking	cell movement of leukocytes	1.82E-04	2.64	*C6*, *CCL20*, *CCR3*, *CSF2*, *DRD3*, *GRP*, *HP*, *IL20*, *IL22*, *IL37*, *IL6*, *LBP*, *RETN*, *TREML2*
	cell movement of myeloid cells	6.05E-05	2.37	*C6*, *CCL20*, *CCR3*, *CSF2*, *GRP*, *HP*, *IL20*, *IL22*, *IL6*, *LBP*, *RETN*, *TREML2*
	cell movement of granulocytes	1.04E-03	2.28	*CCR3*, *CSF2*, *GRP*, *IL20*, *IL22*, *IL6*, *LBP*, *TREML2*
Cellular Movement, Hematological System Development and Function, Immune Cell Trafficking, Inflammatory Response	cell movement of phagocytes	3.28E-04	2.42	*C6*, *CCL20*, *CCR3*, *CSF2*, *GRP*, *HP*, *IL20*, *IL6*, *LBP*, *RETN*, *TREML2*
	cell movement of neutrophils	5.41E-03	2.41	*CSF2*, *GRP*, *IL20*, *IL6*, *LBP*, *TREML2*
	chemotaxis of granulocytes	5.23E-04	2.35	*CCR3*, *CSF2*, *GRP*, *IL20*, *LBP*, *TREML2*
	chemotaxis of myeloid cells	1.88E-04	2.15	*CCL20*, *CCR3*, *CSF2*, *GRP*, *HP*, *IL20*, *LBP*, *TREML2*
	chemotaxis of neutrophils	1.62E-03	2.15	*CSF2*, *GRP*, *IL20*, *LBP*, *TREML2*
	chemotaxis of leukocytes	2.79E-05	2.14	*CCL20*, *CCR3*, *CSF2*, *GRP*, *HP*, *IL20*, *IL22*, *IL6*, *LBP*, *TREML2*
Cellular Movement, Immune Cell Trafficking	leukocyte migration	6.68E-05	2.55	*C6*, *CCL20*, *CCR3*, *COCH*, *CSF2*, *DRD3*, *GRP*, *HP*, *IL20*, *IL22*, *IL37*, *IL6*, *LBP*, *OLR1*, *RETN*, *TREML2*
Developmental Disorder	Hypertrophy	5.84E-03	2.35	*CSF2*, *GAL*, *GRP*, *IL6*, *RRAD*, *SOST*, *TNNT2*, *WNT7A*
Free Radical Scavenging	synthesis of reactive oxygen species	4.37E-04	2.19	*CCR3*, *CSF2*, *HBA1*/*HBA2*, *HP*, *IL24*, *IL6*, *OLR1*, *PEBP4*, *RETN*, *TREML2*
Hematological System Development and Function, Tissue Morphology	quantity of blood cells	3.84E-03	2.31	*C6*, *CCL20*, *CCR3*, *CD79B*, *CSF2*, *GAD2*, *HBA1*/*HBA2*, *IGFBP1*, *IL13RA2*, *IL22*, *IL6*, *IL9R*, *MMP13*, *SPTA1*
Inflammatory Response	inflammatory response	1.38E-05	2.59	*C6*, *CCL20*, *CCR3*, *CSF2*, *DRD3*, *GAL*, *GRP*, *HP*, *IL20*, *IL22*, *IL24*, *IL37*, *IL6*, *LBP*, *OLR1*, *TREML2*
Lipid Metabolism, Molecular Transport, Small Molecule Biochemistry	concentration of lipid	3.37E-04	2.02	*CRHR2*, *CSF2*, *FGFR4*, *GAD2*, *GAL*, *GRP*, *HP*, *IL6*, *LBP*, *MGAT3*, *OLR1*, *RETN*, *RRAD*, *SOST*
	secretion of lipid	3.43E-04	2.00	*CSF2*, *DRD3*, *GAL*, *GRP*, *IL6*, *RETN*
Lipid Metabolism, Small Molecule Biochemistry	synthesis of lipid	3.48E-03	2.40	*CCR3*, *CRHR2*, *CSF2*, *FGFR4*, *FOSL1*, *GAD2*, *GRP*, *IL24*, *IL6*, *OLR1*, *RETN*
Molecular Transport	secretion of molecule	7.96E-05	2.32	*ATP4B*, *CRHR2*, *CSF2*, *DRD3*, *GAL*, *GRP*, *IL6*, *OLR1*, *RETN*, *SLC26A4*, *SYN1*, *WNT7A*
	transport of molecule	1.18E-03	2.21	*ACTN2*, *ATP4B*, *ATP6V1G2*, *CRHR2*, *CSF2*, *DRD3*, *GAL*, *GRIA2*, *GRIK2*, *GRP*, *HBA1*/*HBA2*, *IL6*, *KCNH4*, *LBP*, *OLR1*, *RETN*, *SLC26A1*, *SLC26A4*, *SYN1*, *WNT7A*
Organismal Development	formation of vessel	4.01E-04	2.00	*CRHR2*, *CSF2*, *IL6*, *OLR1*, *OPTC*

^a^activation z-score (|z-score| > 2)

^b^non-redundant TopDEGs (|log_2_| FC > 1)

Among S hosts, seven functions were predicted as activated according to the same criteria (Table 3). We also identified anti-tick related functions as cellular movement (except for granulocytes), cell adhesion, leukocyte immune response, and quantity of calcium.

**Table 3:** Functional enrichment of non-redundant differentially expressed genes presenting higher variation in the skin of tick-susceptible Braford cattle after tick infestation

Category	Function Annotation	*p*-value	z-score^a^	Genes^b^
Cell Signaling, Molecular Transport, Vitamin and Mineral Metabolism	quantity of Ca^2+^	1.74E-03	2.17	*CCL3*, *CXCL11*, *GCGR*, *TAC1*, *TNFRSF9*, *TNFSF11*
Cell-To-Cell Signaling and Interaction, Hematological System Development and Function, Immune Cell Trafficking	adhesion of immune cells	6.86E-04	2.15	*CCL3*, *CXCL11*, *FCAR*, *TAC1*, *TNFRSF9*, *TNFSF11*
Cell-To-Cell Signaling and Interaction, Inflammatory Response	immune response of leukocytes	2.44E-03	2.20	*CCL3*, *FCAR*, *TNFRSF9*, *TNFSF11*, *TREM2*
Cellular Movement, Hematological System Development and Function, Immune Cell Trafficking	cell movement of leukocytes	9.12E-04	2.41	*CCL3*, *CXCL11*, *DMBT1*, *FCAR*, *TAC1*, *TG*, *TNFRSF9*, *TNFSF11*, *TREM2*
	cell movement of antigen presenting cells	9.33E-05	2.17	*CCL3*, *CXCL11*, *DMBT1*, *FCAR*, *TAC1*, *TNFRSF9*, *TREM2*
Cellular Movement, Hematological System Development and Function, Immune Cell Trafficking, Inflammatory Response	cell movement of phagocytes	3.56E-04	2.25	*CCL3*, *CXCL11*, *DMBT1*, *FCAR*, *TAC1*, *TNFRSF9*, *TNFSF11*, *TREM2*
	cell movement of macrophages	1.14E-03	2.22	*CCL3*, *DMBT1*, *FCAR*, *TNFRSF9*, *TREM2*

^a^activation z-score (|z-score| > 2)

^b^non-redundant TopDEGs (|log_2_| FC > 1)

The over-representation of molecular functions evaluated for all annotated DEGs, and the statistical values and symbols which represent the protein classes are presented in Fig. 1d and e. In the intergroup comparisons (Fig. 1d), DEGs from R hosts were enriched (*p* < 0.05) for two protein classes, enzymes and “other” for proteins that do not belong to any other class listed, both before (R*pre*) and after (R*post*) tick infestation. In the intragroup comparisons (Fig. 1e), S hosts showed over-representation of two protein classes after tick infestation (S*post*) not represented in R: receptors and transcription factors (TFs).

Next, functional enrichment by ontology was applied to identify over-represented processes and pathways among TopDEGs from intragroup comparisons, looking for potential anti-tick responses differently represented between hosts. Enrichment for TopDEGs included immune and inflammatory processes common to both hosts and others exclusive to R (Additional file 3: Table S3A). Leukocyte chemotaxis, T helper cell 17 (Th17)-signaling and Jak-STAT pathway represent enriched processes for TopDEGs from R and S hosts after tick infestation, whereas proteolysis in connective tissue degradation and extracellular matrix (ECM) remodeling, inflammation by cytokines signaling and platelet-endothelium-leukocyte interactions were enriched only for R.

In the leukocyte chemotaxis process network, C-C motif chemokine ligand 13 (*CCL13*) gene was up-regulated in both phenotypic classes, although its receptor chemokine C-C motif receptor 3 (*CCR3*) gene was concomitantly up-regulated only in R hosts. Moreover, only R hosts showed two types of cytoskeletal proteins coding genes, tubulin alpha and actinin alpha, as TopDEGs. The lymphocyte attractant *CCL20* gene was strongly down-regulated in R hosts, whereas *CCR1* was TopDEG only in S, as well as its ligand, the macrophage inflammatory protein 1 (*MIP-1 alpha*). Finally, S hosts showed over-expression of the interferon-inducible T-cell alpha chemoattractant (*I-TAC*) gene after tick infestation.

TopDEGs from both hosts were also committed to the Th17-derived cytokines immune process (Additional file 3: Table S3A) with some differences. R hosts over-expressed matrix metallopeptidase 9 (*MMP9*), granulocyte-macrophage-colony-stimulating factor (*GM-CSF*), tissue inhibitor of metalloproteinase 1 (*TIMP1*), interleukin 6 (*IL6)* and interleukin 22 (*IL22*) transcripts that code for proteins involved in response to IL17 as well as in the differentiation of the Th17 cell. Regarding S hosts, we found the over-expression of the cytokine receptor activator of nuclear factor kappa-B ligand (*RANKL*) transcript and the absence of mRNAs corresponding to critical cytokines coding genes in skin inflammation, such as *IL6* and *IL22*.

The Janus kinase-signal transducers and activators of transcription (Jak-STAT) pathway involves signaling cascades that transmit extracellular signals to target genes in the nucleus. Overall, both R and S hosts showed increased gene expression of external stimuli signaling, mainly represented by cytokines, which leads to the activation of this pathway in response to tick infestation. However, S hosts showed an additional inhibition of ciliary neurotrophic factor (*CNTF*) and suppressor of cytokine signaling 2 (*SOCS2*) genes.

Connective tissue degradation and skin ECM remodeling were enriched only for R hosts since genes such as *MMP9*, *TIMP1*, kallikrein 1 (*KLK1*) and *KLK6* presented low FC in S. Additionally, target proteins coding genes were down-regulated in R hosts, such as collagen I, collagen III, lumican, and osteonectin. Overall, R hosts showed more TopDEGs (Additional file 3: Table S3A) related to these two processes after tick infestation when compared to S.

Finally, the TopDEGs from R hosts enriched inflammation by IL6-signaling with up-regulation of genes participating in this signaling pathway beyond *IL6* itself*,* such as acute-phase protein coding genes such as *SAA3*, haptoglobin (*HP*), and lipopolysaccharide-binding protein (*LBP*), and the acquired-immunity cytokine *IL22* (Additional file 3: Table S3A).

**Fig. 2:**
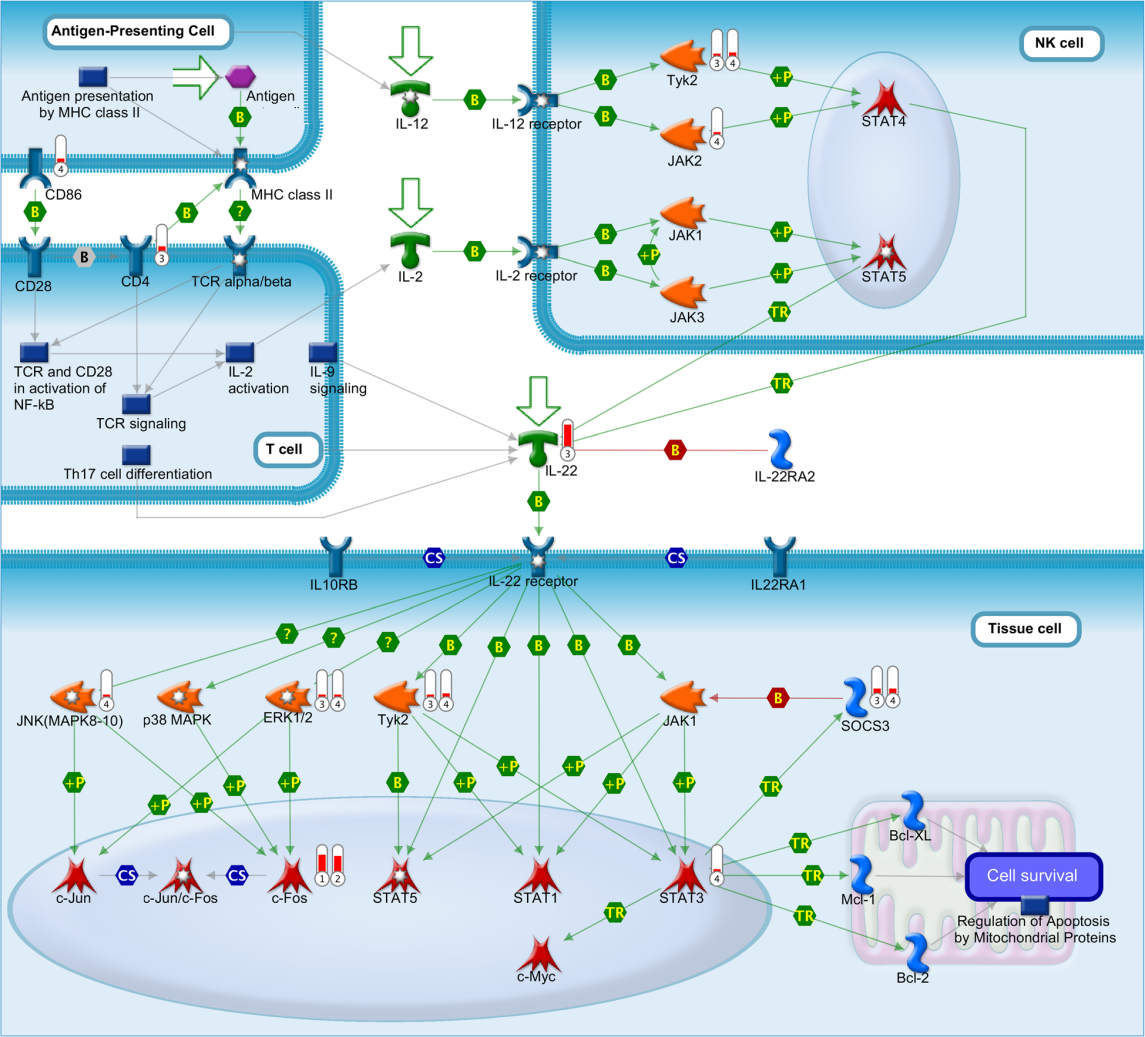
Interleukin 22 (*IL22)*-signaling pathways in different cell types. *IL22*, produced mainly by immune CD4^+^ T cells, acts upon many different tissue cells, with a key role in the skin, digestive, and respiratory tracts. *IL22* acting via its heterodimeric receptor induces activation of many kinases (via MAPs and Jak*-*STAT pathway) and transcription factors compromised with the expression of pro-inflammatory cytokines. DEGs from inter- (R*pre* vs. S*pre* (1) and R*post* vs. S*post* (2)) and intragroup (R*post* vs. R*pre* (3) and S*post* vs. S*pre* (4)) comparisons and their respective modulation are shown aside the network objects. Values of expression are represented by colored bars in thermometer-like icons. The red bar represents up-regulation. Network object symbols follow the Metacore® reference guide according to the protein class (https://portal.genego.com/legends/MetaCoreQuickReferenceGuide.pdf), and their names may occasionally differ between tables and the figure due to the MetaBase®

The enrichment based on pathways pointed to ECM remodeling and some cytokines for TopDEGs from both hosts, but indicated the putative participation of T cells only in R (Additional file 3: Table S3B). In fact, through pathway analysis, DEGs from R hosts supported the participation of T CD4^+^ cell subtypes, including IL22-signaling pathway (Fig. 2). Genes, such as *CD4*, *IL22*, and *c-Fos*, were modulated only in R. Others, as tyrosine kinase 2 (*Tyk2*), *ERK1/2* and *SOCS3*, were modulated in both hosts, while only S hosts showed modulation of *STAT3*. All DEGs in this pathway were up-regulated, including the effectors (as *c-Fos*) and the regulators (as *SOCS3*).

**Fig. 3:**
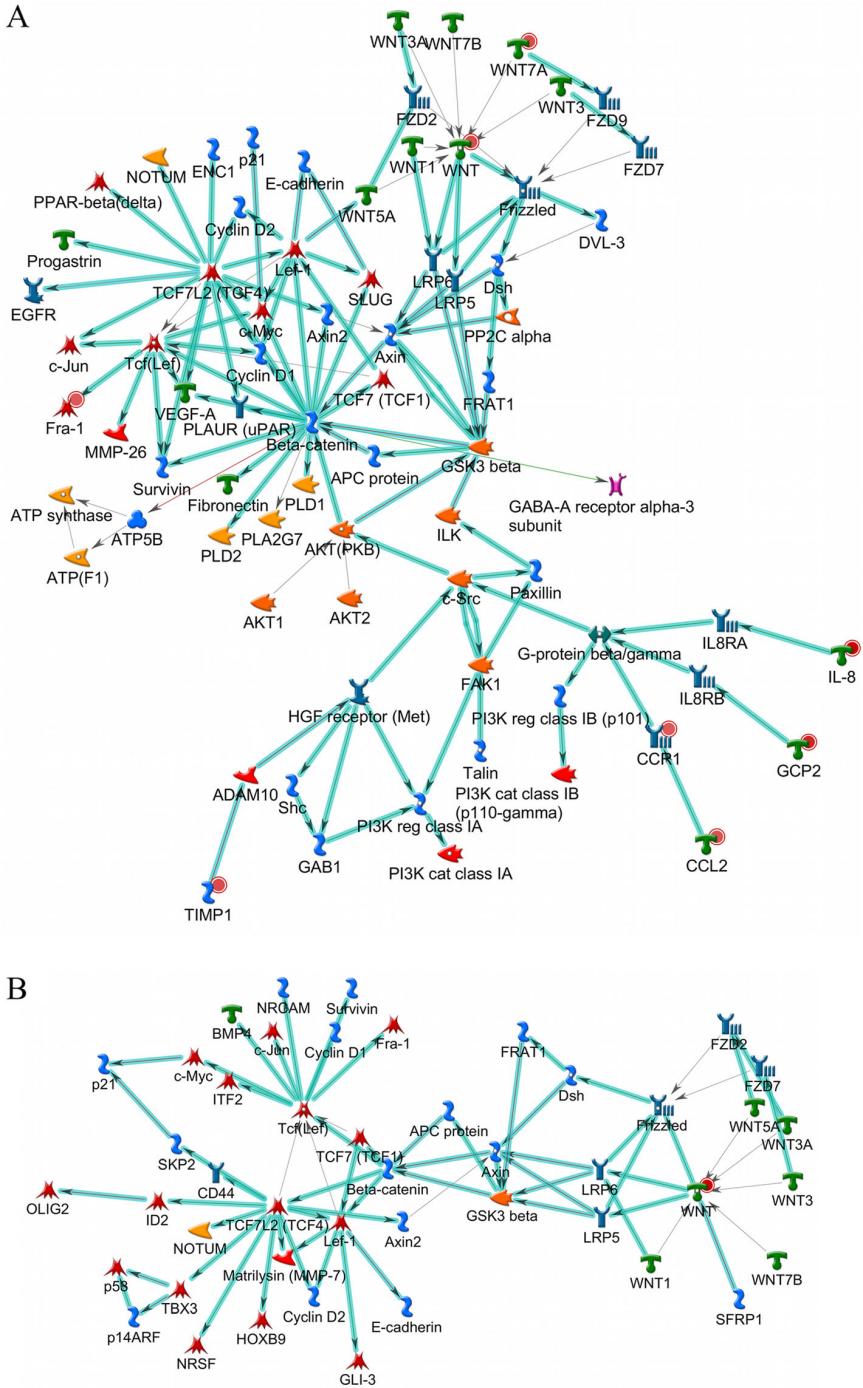
Wingless (WNT*)-*signaling pathway as a central way to Braford anti-tick resistant (R) response. (**a**) The canonical WNT-family pathway was predicted for the TopDEGs from R hosts after tick infestation, highlighted in cyan. Red circles represent over-expression: higher the value, stronger the intensity of red. (**b**) Genes prospected from genome-wide association study also indicate the WNT-signaling pathway as the most relevant in resistance. The red circle represents the prospected gene. Network object symbols follow the Metacore® reference guide according to the protein class (https://portal.genego.com/legends/MetaCoreQuickReferenceGuide.pdf), and their names may occasionally differ between tables and the figure due to the MetaBase®

**Network Analysis.** The WNT-signaling pathway, including WNT*/β-*catenin dependent, was the most relevant network predicted for R hosts (Fig. 3). This pathway is strongly supported by TopDEGs, especially the *WNT7A* gene (Fig. 3a), and also included DEGs (Additional file 2: Table S2). Among them, the TF-coding gene lymphoid enhancer-binding factor 1 (*LEF1*) and the anti-apoptotic and inflammatory gene B-cell lymphoma 9 protein (*bcl9*) were up-regulated in R, whereas plasminogen activator, urokinase (*PLAU*), PLAU receptor (*PLAUR*), low-density lipoprotein receptor-related protein 12 (*LRP12*) and members of the fibroblast growth factor (*FGF*) family were up-regulated in both hosts. On the other hand, the receptor coding gene *LRP2* was down-regulated only in S. There was no evidence of the involvement of WNT-Ca pathway (or non-canonical WNT pathway) in resistance. The complete list of predicted networks for R hosts is shown in Additional file 4: Table S4, and includes skin biology, cell surface receptor signaling, response to an endogenous and external stimulus, inflammatory and defense pathways.

Network analysis was next applied to a subset of genes, based on LD (linkage disequilibrium) information from this population [39], within 200 kb genomic regions from each side of TagSNPs explaining up to 20% of the resistance variation in a GWAS [36], similar to the approach described by Mota et al. (2017) [40]. The canonical WNT network (β-catenin dependent) was predicted as the most relevant pathway for those genes (Fig. 3b and Additional file 5: Table S5), corroborating the involvement of this pathway in resistance and supporting our finding of *WNT7A* as TopDEG in the intergroup comparisons (Table 1). The complete list of predicted networks for genes prospected from the GWAS is shown in Additional file 5: Table S5.

**Fig. 4:**
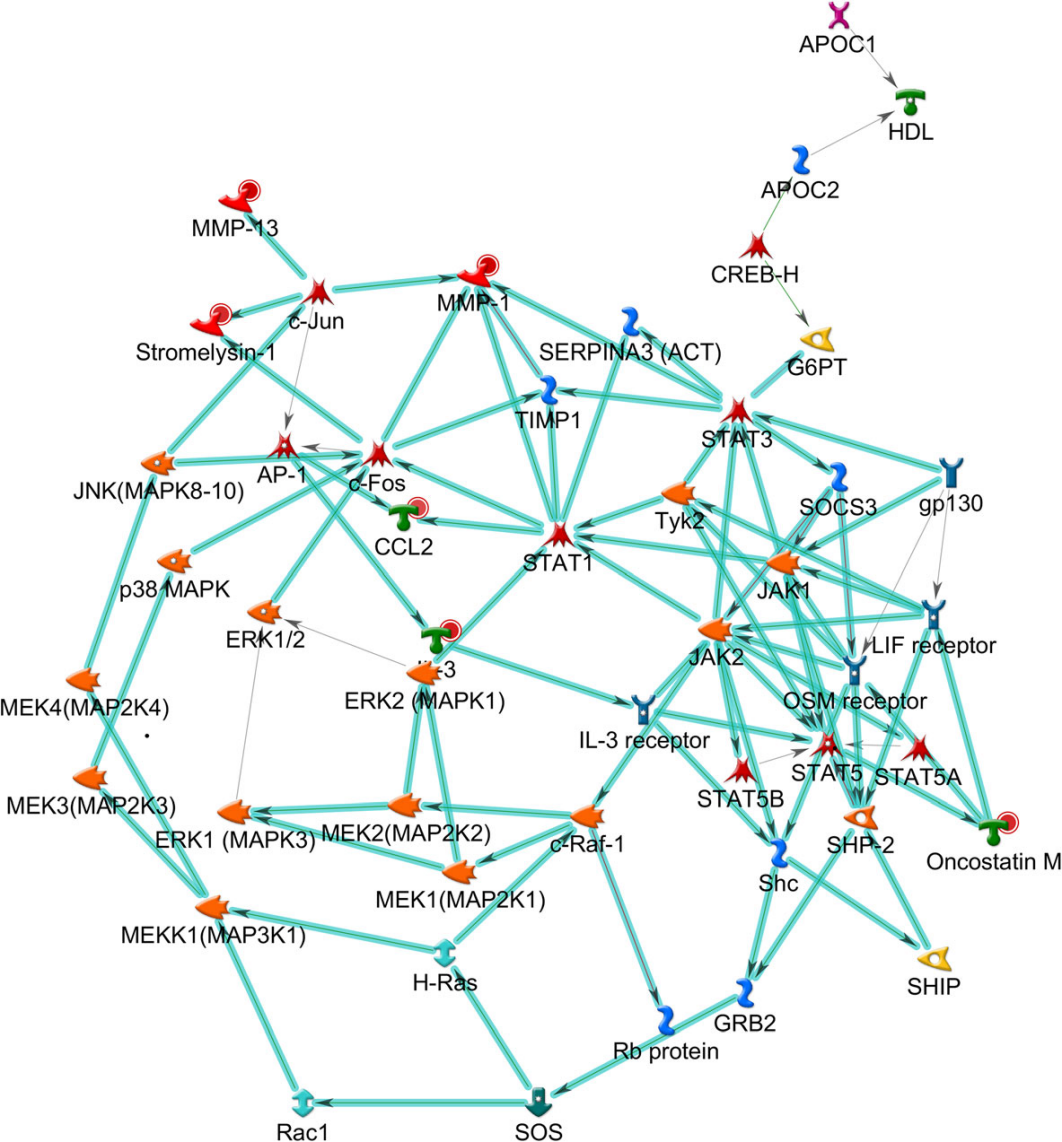
Regulation of immune response as the most relevant pathway in the infested susceptible (S) skin. Regulation of immune response was predicted for the TopDEGs from S hosts after tick infestation, highlighted in cyan. Red circles represent over-expression: higher the value, stronger the intensity of red. Network object symbols follow the Metacore® reference guide according to the protein class (https://portal.genego.com/legends/MetaCoreQuickReferenceGuide.pdf), and their names may occasionally differ between tables and the figure due to the MetaBase®

On the other hand, the most relevant network for S hosts involved *MMP1*, oncostatin M (*OSM*), stromelysin-1, *CCL2*, and *IL3* genes (Fig. 4 and Additional file 6: Table S6) and was related mainly to the regulation of immune responses. Other networks also pointed to immune-related processes, but with low connectivity scores.

Finally, the prediction of potential TFs underlying the regulation of TopDEGs from R hosts pointed to Ap1 family members, such as *c-Fos*, *JunB*, *Fra1*, *FosB*, and *Fra2* (Additional file 7: Table S7). *Fra1* and *Fra2* were also TopDEGs. As the TF lists were manually curated according to the recently published bovine TFs compendium [41], only TFs found in cattle were shown. For S hosts, a nuclear factor kappa B (*NF-kB*) family member, the NF-kB subunit p65 (*RelA*), was the most relevant TF predicted. Although some Fos family members also appeared in the list, such as *c-Fos* and *c-Jun*, the NF*-*kB family was represented by three other members. Of which, the most relevant TFs are as follows: *NF-kB* subunit p50 (*NFKB1*), *cRel* and *RelB*. The TopDEG TFs from S hosts were the nuclear factor, erythroid 2 (*NF-E2*), another NF*-*kB family member, CCAAT/enhancer-binding protein (*C/EBPE*) and paired box 8 (*PAX8*), which were classified below the 60th position according to their *p*-value and z-score (Additional file 8: Table S8).

## Discussion

### Differential gene expression analysis

The mechanisms underlying resistance and susceptibility against ticks of phenotypically divergent Braford hosts were addressed using RNA-Seq in cattle skin. With this approach, the extension of genetic modulation induced by ticks could be better examined in terms of differential gene expression and their collective participation in resistance (and susceptibility) at system biology levels. The gene expression profiles found before challenge (*pre*) could be partially explained by the immunity previously acquired by hosts, since they were not *naive*. Additionally, 24 h after the fourth tick infestation (*post*), we expected to see both innate and acquired immune responses, as already demonstrated for re-infested animals in skin-level studies of gene expression [14, 22, 23, 28].

Through the intergroup comparisons, we investigated the gene expression profiles of R and S hosts, looking for candidate genes that would explain phenotypic differences, according to the infestation challenge: R*pre* vs. S*pre* (1) and R*post* vs. S*post* (2). Both differential expression analyses revealed highly redundant DEG lists, suggesting that mechanisms of resistance in Braford cattle were present before challenge. Natural infestations before the first skin sampling, the time of skin sampling after infestation (24 h) or even the absence of the tick attachment site at the biopsy could contribute to these findings. Almost all annotated DEGs were classified as TopDEGs with biological functions correlated to cattle resistance against ticks. Overall, they were involved in hemostatic and immune mechanisms, many of them already associated with tick infestation, such as coagulation, iron metabolism, and inflammation [42–44]. It is conceivable that the modulation of these functions could help hosts achieve a better initial cellular response against tick antigens when tick feeding starts.

*AWAT1*, which had the highest variation in the intergroup comparisons, encodes an enzyme that acts in lipid metabolism and sebum composition in the skin [45]. Lipid metabolism has a role in inflammation control [46] and semiochemistry [47], and probably also in anti-tick resistance [24]. The tachykinin family (represented by *TACR2* gene) was widely associated with inflammatory response, playing a role in wound healing and immune cell differentiation [48, 49]. Seric amyloid (SAA) family members were associated with anti-tick resistant response [50], and *SAA3* expression was up-regulated in R hosts in addition to the intragroup comparison (Additional file 2: Table S2). Amine oxidase copper containing 3 (*SAO*) gene product is committed to leukocyte trafficking [51, 52], a function already associated with anti-tick responses [53], as well as in vascularization and tissue organization. Genes, such as bolA family member (*BOLA*) and tetratricopeptidase repeat domain 36 (*TTC36*), are related to immune mechanisms, including antigen processing and presentation. Moreover, genes involved in skin organization as collagen type XI alpha 1 (*COL11A1*), laminin alpha 1 (*LAMA1*) and agouti signaling protein (*ASIP*) may also contribute physically to tick bite healing, unsuccessful attachment, and establishment of the feeding site. Finally, the ectonucleotide pyrophosphatase/phosphodiesterase 3 (*ENPP3*) gene encodes an enzyme committed to the hydrolysis of extracellular nucleotides, also known as *CD203c.* This enzyme is better described as an activation marker of basophils, a cell type associated with acquired anti-tick resistance [25, 54, 55].

Genes modulated only before infestation (*Rpre*) are probably involved in primary cellular functions that may prepare R hosts to promptly react to infestation. *DPYSL4* gene is described as a target of p53 in the apoptotic response to DNA damage [56]; once up-regulated, it could contribute to the apoptosis in the tick bite site. *MTHFS* is associated with folate turnover rate and depletion [57], and folate is essential for healthy skin [58]. Finally, over-expression of *UTS2R,* even in the absence of active infestation, suggests better control of vascular dynamics and osmoregulation in R hosts.

On the other hand, genes modulated only in R*post* may indicate putative mechanisms of protective response induced by the presence of ticks. Although the proteins encoded by genes such as *UPK3A*, *TET1* and *RBP1* do not play a direct role in immune cell functions, they indirectly help immune response. The function of *UPK3A* gene product has been linked to epithelial differentiation [59]. *TET1*, inhibited in R*post*, encodes a transcription repressor of a subset of genes, including interleukin 1 beta (*IL1b*), *IL8*, *IL23*, intercellular adhesion molecule 1 (*ICAM1*) and chemokine C-X-C motif ligand 1 (*CXCL1*) [60]. Most of these genes, modulated by *TET1* expression, were up-regulated in R*post* (Additional file 2: Table S2). *RBP1*, also up-regulated in R hosts, encodes the carrier protein of retinol (vitamin A alcohol) transport, making it accessible in peripheral tissues. Besides the role in epithelial tissues, the hormone-like properties of vitamin A binding to nuclear hormone receptors retinoic acid receptors (*RAR*) and peroxisome proliferator-activated receptor (*PPAR*) affect immunity [61, 62]. This gene was up-regulated in R*post* compared to S*post* and down-regulated in S*post* compared to S*pre*, suggesting its involvement in tick resistance. Although the *MYADML* family is still incompletely annotated, some products have been described as modulators of cell spread and migration to the skin [63]. Three members of this family were modulated in R*post* with contrasting modulation (log_2_ FC -5.88, − 2.4 and 6.33; Table 1), which could indicate antagonistic effects regarding response to tick infestation.

In the intragroup comparisons, R*post* vs. R*pre* (3) and S*post* vs. S*pre* (4), we were looking for genes elicited by tick challenge, according to the resistance group studied. They revealed extensive gene lists, with thousands of DEGs broadly distributed over the genome, showing both redundancy and high correlation between hosts DEGs as well as non-redundant DEGs for R or S hosts (Additional file 2: Table S2). Critical functions against infestation, such as immune responses, coagulation, vascularization, and ion transport, were shared between hosts. This indicates that tick infestation caused a comprehensive response in Braford skin, regardless of being an R or an S host. On the other hand, non-redundant DEGs with potential anti-tick functions may represent mechanisms of response that contribute to differentiating hosts regarding resistance. This hypothesis was supported by the activation of critical functions in R hosts with massive participation of all immune cells (Table 2), which had the potential to protect them. Among S, cellular activation was not predicted for granulocytes (Table 3), an important cell type for tick resistance [19, 23, 54]. Overall, the data suggest the relevance of cytokines in R skin reactions. The inhibition of grooming predicted for R hosts is probably due to the time of skin sampling (24 h) when larvae had already been rejected [64]. Although very little is known about this, grooming behavior is considered to have an impact on tick load [15] and probably to ectoparasites other than ticks as well.

More than a hundred immune-related genes encoding cytokines, chemokines, CD markers, acute phase proteins, complement proteins, integrins, and TFs were found (Additional file 2: Table S2). Our results revealed novel DEGs associated with resistance/susceptibility, DEGs that support previous results and predictions, and DEGs that diverge with authors comparing gene expression between taurine and zebuine breeds [14, 21–25], within other cattle breeds [28–31] or murine models [37, 65]. According to Piper and colleagues [21, 23], *CXCL2* and *CCL2* were modulated only in S hosts; however, we found them also modulated in R. *CCR1* and *IL2R* were described as modulated in R and *CD14* in S [26], in contrast with our results where *CCR1* and *CD14* were modulated in both hosts, and *IL2RG* up-regulated only in S. *IL8* was up-regulated in both R and S, but was previously described as down-regulated in R [33] and up-regulated in S [23]. *IL13RA1* was described as up-regulated in S and down-regulated in R [28], whereas we found *IL13RA1* to be up-regulated in S and *IL13RA2* to be up-regulated in R. Regarding T cell participation in resistance [14, 22, 23, 25, 27, 30, 31], *CD4* was up-regulated only in R hosts, in agreement with previous results. These inconsistencies may be the result of a myriad of factors, including sensibility of different techniques for gene expression analysis, experimental design, epistatic interactions with the genetic background and environmental effects, and indicate the need for extensive research on specific tick-host relationships.

### Enrichment analysis (EA) based on functional ontologies

In the intergroup comparisons, similar protein classes were enriched among TopDEGs, although the number of TopDEGs was insufficient for network analysis. In the intragroup comparisons, S hosts showed more enriched protein classes than R. Functional enrichment was applied to TopDEGs (Additional file 3: Table S3) as this analysis is a powerful tool to determine the relevance of a gene in the context of a pathway. Some processes, which can be crucial to managing the interference of tick in the skin immune biology were over-represented in both hosts, including inflammation, chemotaxis, and immune response. Connective tissue degradation, matrix remodeling and immune process networks found only in R hosts likely indicate mechanisms they use to respond to ticks which are not elicited in S.

Even though both hosts had enriched leukocyte chemotaxis, differences between R and S in the modulation of this network may contribute to susceptibility (Additional file 3: Table S3A). For example, *CCR3* was a TopDEG up-regulated only in R, although the corresponding ligand *CCL13* was up-regulated in both phenotypes, thus suggesting differences in CCL13 effectiveness. CCL13 is involved in chemotaxis of eosinophils, basophils, and Th1 cells, all of which were previously associated with resistance [19, 23, 54]. The modulation of cytoskeletal proteins may also contribute to more efficient chemotaxis. On the other hand, the down-regulation of *CCL20* in R hosts could indicate inhibition of the recruitment of lymphocyte subpopulation to the resistant skin [66]. The over-expression of *MIP-1 alpha* and *I-TAC* in S hosts are other examples: the former encodes a chemokine involved in inflammation, especially in attracting neutrophils and macrophages, which are known to have their functions impaired by tick saliva [43, 67, 68]; I-TAC, a chemoattractant of activated lymphocytes produced mainly by basal keratinocytes [69], did not find its receptor coding gene C-X-C motif chemokine receptor 3 (*CXCR3*) modulated, which was expected in activated T cells responding to infestation. Although tick infestation also elicits a cellular response in S hosts, it may attract less efficient cells to their skin.

Additionally, Th17-derived cytokines network was also over-represented among TopDEGs from both hosts (Additional file 3: Table S3A). Th17 cells belong to a distinct subset of CD4^+^ Th lymphocytes characterized as preferential producers of IL17A, IL17F, IL22, and, in humans, IL26. The receptors for IL17 and IL22 are broadly expressed in various epithelial tissues. The effector cytokines released from Th17 cells affect different cellular populations in inflammatory sites, mediating host defense through the activation of several signaling pathways. The participation of the IL17 family in cattle anti-tick response has already been proposed [25].

Finally, Jak-STAT pathway was also enriched for both hosts (Additional file 3: Table S3A). In this pathway, membrane-associated tyrosine kinases lead to the phosphorylation of signaling proteins and transcription factors such as STATs, regulating the expression of genes involved in the inflammatory response, cell survival, and cell cycle in response to numerous cytokines and growth factors. S hosts showed down-regulation of *CNTF* whose product, in the nervous system, appears to protect tissue during inflammation, and *SOCS2*, a Jak*-*STAT regulator coding gene whose product interacts with major molecules of signaling complexes to block further signal transduction.

Connective tissue degradation and ECM-remodeling networks were over-represented only among TopDEGs from R. These are two particularly important processes which require the over-expression and activation of proteinases able to destroy the connective tissue, allowing non-resident inflammatory cells arrival to the skin [23]. Most proteolytic enzymes are MMPs, and proteins including disintegrin and metallopeptidase domain (ADAMs), KLKs, elastases, trypsin, and others also participate. The MMPs comprise a family of enzymes that can collectively degrade all components of the ECM. They are the main proteases involved in ECM remodeling and are negatively regulated by TIMPs. The ability to modulate the skin immune-biology to impair tick success may represent a great difference between R and S hosts.

Inflammation via IL6-signaling was also over-represented only in TopDEGs from R hosts, in contrast with previous reports in which both R and S hosts show up-regulation of *IL6* [25]; it is also reported in mice-infested skin, a model considered susceptible to tick [65]. This cytokine is secreted during inflammation by macrophages, endothelial cells, and fibroblasts. Its primary effect is to induce the secretion of acute-phase proteins, whose role as mediators of inflammatory responses has already been associated with resistance against ticks [24, 50]. IL6 also participates in the differentiation of some cell types, particularly in inducing B lymphocytes to differentiate into plasma cells; it can also lead to anti-apoptotic effects [70].

Given this, the up-regulation of *IL6* together with many acute-phase protein coding genes and *IL22* may create a favorable scenario for the maintenance of healthy skin barrier, since IL22 plays a critical role in skin immunity, inflammation, and repair [71]. Th22 participation in tick resistance was supported by the pathway analysis using TopDEGs from R hosts. Previous works have demonstrated the massive infiltration of CD4^+^ cells in the skin of infested animals as well as an increased number of these cells in the bloodstream and lymph nodes [14, 22, 26, 27, 30, 31]. IL22 is a member of the IL10 family of cytokines [72] that acts via a heterodimeric receptor complex (IL22 receptor) consisting of the subunits IL22RA1 and IL10RB (Fig. 2). IL22 binding to IL22 receptor activates the Jak*-*STAT pathway, inducing the phosphorylation of JAK1, Tyk2*,* and of the transcription factors STAT1, STAT3, and STAT5. IL22 also activates three major mitogen-activated protein (MAP) kinase pathways, c-Jun NH2-terminal kinase (JNK), p38 MAPK and extracellular signal-regulated kinase (ERK1*/2*), which are possibly required for activation of c-Jun/c-Fos to elicit the expression of pro-inflammatory cytokine genes [73, 74]. The primary sources of IL22 are immune cells, whereas its receptor is found in non-hematopoietic cells, especially at outer body barriers such as skin and the digestive and respiratory tracts [71, 75, 76]. Many genes in the Th22 pathway were DEGs in R hosts after infestation, including *IL22* itself and other genes involved in response to this cytokine in tissue cells, as the tyrosine kinases and *c-Fos* (Fig. 2). Additional file 2: Table S2 illustrates substantial evidence for the differential activation of IL22-mediated immune response in R hosts, with the up-regulation of *IL20*, *IL22*, *HP*, *JUN*, *FOSL1*, *IL9R*, *MMP13*, *CCR3*, colony-stimulating factor 2 (*CSF2*) and *IL6* in R but not in S, suggesting a putative role of this immune mechanism in resistance against ticks in Braford cattle. S hosts also showed some DEGs in this pathway, though neither *IL22* was up-regulated, nor *IL6*, whose product acts in the Th22 cell differentiation [71]. Robbertse and colleagues (2018) [19] suggested the modulation of cytokines, such as IL6, IL13, IL5, chemokines, and of receptors as CCL2 and CCR1, acute phase proteins, cell adhesion molecules, CD4 and CD14 molecules, with the resulting attraction of T and B lymphocytes and granulocytes to the skin, to be associated with resistance. Our results partially agree with the predictions of these authors, since the modulation of some DEGs was redundant between R and S Braford hosts.

### Network analysis

This analysis was carried out to identify the central mechanisms and to better explain contrasting host phenotypes based on their differential gene expression. It showed partially overlapping size-limited sub-networks, expanded from the TopDEGs lists, giving preference to objects with more connectivity considering that highly connected genes tend to be involved in similar biological functions.

WNT-signaling, the most relevant pathway predicted for R hosts (Additional file 4: Table S4 and Fig. 3a), is an evolutionarily conserved pathway that regulates the key process of organogenesis during embryonic development, cell fate determination, cell migration and polarity [77]. Recently, this pathway has also been associated with functions in immune response [78, 79] and skin biology as well [80–82]. Its effects on inflammation can be both pro- and anti-inflammatory, depending on the stimulus, cell type, activation context, and its crosstalk with other signaling pathways [78, 83]. It becomes inflammatory in infections with pathogenic bacteria and in inflammatory bowel diseases [78, 84]. However, it has also been associated with the induction of tolerogenic dendritic cells (DCs), which participate in immune response regulation mainly through the inhibition of Th1/Th17 cytokines [79]. It also participates in skin wound healing, through its ability to induce self-renewal and proliferation of skin stem cells [80, 82]. Finally, this pathway plays a critical role in many physiological processes of the skin from the earliest stages of development to postnatal control of hair cycling, inter-follicular epidermis maintenance as well as hair and skin pigmentation [81]. Furthermore, the deregulation of this pathway has already been associated with skin diseases [81] and host-tick interface [65]. Hair coat morphology and pigmentation were also associated with tick counts [85, 86]. Additionally, the WNT-signaling pathway was predicted in our analysis of genes mined from TagSNPs associated with resistance to ticks in the same breed [36] (Additional file 5: Table S5 and Fig. 3b). The importance of wound healing and structural proteins for tick resistance has been suggested [21, 37, 65]. Our results suggest the participation of WNT pathway in anti-tick resistance through an inflammatory scenario comprising the regulation of cytokines, receptors, matrix proteinases, and transcription factors and, at the same time, through its role in skin healing and remodeling, which hinders tick success.

On the other hand, the most relevant pathway for TopDEGs from S hosts includes *MMP1*, *OSM*, stromelysin-1, *CCL2*, and *IL3* (Additional file 6: Table S6 and Fig. 4), which act on the regulation of immune response. This pathway included molecules related to tissue remodeling, such as OSM, MMPs*,* and TIMPs*,* potentially activating Jak*-*STAT and MAPK signaling cascades [87]. Most of the modulated genes in this pathway were DEGs (not TopDEGs), although Piper and colleagues [23] suggested ticks can more easily modulate the skin of S hosts.

Considering the complexity of host anti-tick resistance, which involves multiple mechanisms of activation and regulation of gene expression to drive the cellular and immune responses, we investigated the role of TFs as key molecules to orchestrate this modulation. Despite the general lack of knowledge concerning the regulation of gene expression in cattle, the TF lists were filtered according to the recently published manually-curated bovine TF compendium [41] to show only bovine TF candidates. For R hosts, Fos family members (*c-Fos*, *c-Jun, Fra1*, and *FosB*) were predicted as the most relevant TFs, with the latest two being TopDEGs (Additional file 7: Table S7), reinforcing the complexity of the regulation of gene expression in R host response against ticks. For S hosts, a nuclear factor kappa B (NF*-*kB) family member was the main TF based on its g-score (Additional file 8: Table S8), although Fos family members were also identified with lower relevance. Even though we extensively analyzed the data integrating different approaches, it was impossible to cover all aspects of the complexity of the host anti-tick response in this study. Experiments designed to compare different skin biopsies, using co-expression analysis and proteomic approaches could certainly drive the next steps of the investigation of this complex trait.

## Conclusions

In the present study, we investigated the response to tick infestation using RNA-Seq data and identified putative genetic mechanisms differentiating R and S Braford hosts. DEGs included genes already associated with resistance using other strategies but were far from being restricted to them. Beyond immune-related genes as cytokines, chemokines, integrins, immunoglobulin superfamily members, acute phase proteins, we found TFs, skin structural, and wound healing related-genes. Collectively viewed, our results provide substantial evidence that R hosts presented an inflammatory response to infestation based on cytokines and the WNT-signaling pathway, potentially impairing tick attachment and feeding success. S hosts seemed unable to assemble an effective anti-tick immunity, even though the infestation also elicited their responses. Our findings shed light on genetic mechanisms and candidate genes underlying tick resistance and susceptibility in Braford cattle.

## Methods

### Animals and skin sampling

Braford heifers, selected from a 974-cohort group belonging to producers affiliated with the Delta G Connection Breeding Program, were segregated into R (*n* = 20) and S (*n* = 19) hosts according to their Estimated Breeding Values (EBVs) and tick counts [88]. Statistic parameter estimates used in animal selection are presented in Additional file 9: Table S9. In addition to having the lower EBVs, the R hosts were always at least one standard deviation (SD) below the cohort average tick count and, when taken the average SD of subsequent counts, they were among the lower 10% of the SD distribution. The same criteria were used to classify as S host, i.e., having the higher EBVs, being at least one SD above the cohort average, and among the 10% lower SD distribution of subsequent counts. These criteria guaranteed R and S hosts had consistently lower and higher tick counts, respectively, within the cohort group. Heifers were transferred from farms to EMBRAPA Pecuária Sul experimental station and maintained for three months in a tick-free pasture receiving an acaricide treatment every two weeks to become non-infested. For the RNA profiling experiment, each animal had four artificial infestations at 14-day intervals, with approximately 20,000 *R. microplus* larvae (obtained from 1 g of eggs) released on the animal’s dorsal line from the neck to the tail insertion. Skin biopsies were collected from each animal using an 8 mm punch before the first infestation (*pre*) and 24 h after the fourth infestation (*post*) without including the tick bite site. The neck area was chosen for sample collection since it was close to the larvae release site and is a frequent place for tick attachment. After the experiment, the sampled heifers were returned to their owner’s farms and reintroduced to their production systems.

### Tick counts

Phenotypic data were collected in four successive infestations over five consecutive days (D19 to D23) post-infestation, considering engorged females bigger than 4 mm which were attached on the left side of the host, as described in [88]. The average number of ticks counted in R hosts used in the present study was significantly (*P* ≤ 0.001) lower (5.48 ticks per body left side) than in S (80.55 per body left side) (Additional file 9: Table S9).

### RNA isolation and sequencing

Skin biopsies were mechanically crushed with 7 mm metallic beads in the TissueLyser equipment (Qiagen), and total RNA harvested using the RNeasy Fibrous Tissue kit (Qiagen). Sequencing libraries were prepared individually using TruSeq RNA Sample Preparation kit v2 guide (Illumina, San Diego, CA) with standard protocols, clustered using TruSeq PE Cluster Kit v3-cBot-HS kit (Illumina, San Diego, CA) and sequenced in a paired-end mode (2 × 100 bp) on a HiSeq1500 equipment (Illumina, San Diego, CA). Reads passing the CASAVA software (Illumina, San Diego, CA) quality controls were used for data analysis.

### RNA-Seq data processing and analysis

Read quality trimming was performed by using SeqyClean v. 1.3.12 [89] to select reads with average Phred score quality (Q) ≥ 30. Quality control was visualized using FASTQC software v. 0.11.1 (http://www.bioinformatics.babraham.ac.uk/pojects/fastqc). The reads were aligned to the bovine reference genome (UMD 3.1, v 75) using TopHat2 [90] following default parameters. Mapped reads were normalized according to the sequencing depth of their group and counted at the gene level using summarizeOverlaps package (May 2014) from R (v. 3.1.1) through “IntersectionNotEmpty” mode.

### Differential gene expression analysis

Differential gene expression analysis was carried out with the edgeR Bioconductor package (v. 3.7.17) [91]. Dispersions were estimated using a generalized linear model (GLM) considering animal, phenotype (R or S) and time (*pre* or *post* infestation). Normalized reads were compared for intergroups: *Rpre* vs. *Spre* (1) and *Rpost* vs. *Spost* (2); and intragroups *Rpre* vs. *Rpost* (3) and *Spre* vs. *Spost* (4). Differential gene expression was tested using the likelihood ratio test corrected for multiple testing by Benjamini-Hochberg method. Genes with significant *p*-value after correcting for multiple testing (FDR < 0.05) were classified as DEGs. TopDEGs were established with |log_2_| FC > 1 and FDR < 0.05. DEGs were annotated based on their Ensembl identifiers using Biomart (v. 2.22.0).

### Enrichment and network analysis

Functional enrichment was performed using the Ingenuity Pathway Analysis (IPA - Qiagen) and MetaCoreTM (Thomson-Reuters) softwares. IPA was used to evaluate the activation/inhibition of non-redundant gene functions of R and S hosts (|z-score| > 2), with positive and negative values indicating, respectively, activation and inhibition of the function. Using MetaCoreTM®, the functional enrichment analysis classified genes according to protein categories and identified the over-represented processes (Process Networks) and pathways (Pathway Maps) among TopDEGs. Network analysis was based on proprietary algorithms to build and analyze partially overlapping size-limited sub-networks (100 network objects), expanded from the initial gene list, giving preference to objects with more connectivity. Beyond the p-value and FDR, two other scores were associated. The z-score considers the number of DEGs represented in the network (saturation), and the g-score represents the saturation of the network with expressed genes considering the number of canonical (classical) pathways used to build it. Since the g-score accounts for a more robust parameter to classify networks, it was chosen to represent them. Network analysis was also applied to genes harboring the TagSNPs selected in previous work [16], where 3545 animals from the same population were phenotyped (ticks counted) and genotyped with the Illumina BovineSNP50 BeadChip. Finally, network analysis was applied to predict TFs considering the differential expression of each TF as DEGs in the data sets.

## Supplementary information

**Additional file 1: Table S1.** Sequencing throughput and mapping statistics for each resistance group. The average, minimum and maximum values of numbers (N) of reads passing filtering and aligned pairs, and the percentage (%) of overall read mapping, multiple alignments, and concordant pair alignment rate are shown. Values were compared inter- and intragroups. Different letters (a and b) represent statistical significance. (XLS 28 kb)

**Additional file 2: Table S2.** Intragroup comparisons of differentially expressed genes in the skin of tick-resistant and -susceptible Braford cattle. DEGs are listed alphabetically with their Ensembl ID and description. The log_2_ FC and the FDR for each DEG are shown in the last two columns, respectively, with the results comparing R*post* vs. R*pre* represented by white cells and S*post* vs. S*pre* represented by gray cells. (XLS 866 kb)

**Additional file 3: Table S3.** Enrichment analysis report of TopDEGs from intragroup comparisons. A) Enrichment by process networks of TopDEGs from resistant (R*post* vs. R*pre*) and susceptible (S*post* vs. S*pre*) hosts. Networks were classified according to the *p*-value, the false discovery rate (FDR), and the number of network objects found in the TopDEGs list (In Data). Total represents the number of total network objects in each network. Unique TopDEGs are shown in bold. B) Enrichment by pathway maps of TopDEGs from resistant (R*post* vs. R*pre*) and susceptible (S*post* vs. S*pre*) hosts. Maps were classified following the same scheme described for networks. (XLS 39 kb)

**Additional file 4: Table S4.** Network prediction for TopDEGs from resistant response to tick infestation. For each network, the key network objects and the GO process associated with them, followed by the number of total and seed nodes, and pathways are shown. The *p*-value, z-score, and g-score classified the networks. (XLS 42 kb)

**Additional file 5: Table S5** Network prediction for genes mined from the TagSNPs associated with resistance to ticks prospected in a genome-wide association study. For each network, the key network objects and the GO process associated with them, followed by the number of total and seed nodes, and pathways are shown. The p-value, z-score, and g-score classified the networks. (XLS 34 kb)

**Additional file 6: Table S6.** Network prediction for TopDEGs from susceptible response to tick infestation. For each network, the key network objects and the GO process associated with them, followed by the number of total and seed nodes, and pathways are shown. The p-value, z-score, and g-score classified the networks. (XLS 40 kb)

**Additional file 7: Table S7.** Transcription factors (TFs) prediction for resistant hosts. Network analysis predicted potential TFs underlying the regulation of gene expression, based on the variation of TopDEGs. The output file was manually-curated, and only TFs found in cattle were classified according to their z-score. TopDEGs TFs were also indicated with their respective modulation and statistical significance. Network object name: network object name in MetaBase®; Actual: number of network objects in the activated dataset(s) which interact with the chosen object; n: number of network objects in the activated dataset(s); R: number of network objects in the complete database or background list which interact with the chosen object; N: total number of gene-based objects in the complete database or background list; Expected: mean value for hypergeometric distribution (n*R/N); Ratio: connectivity ratio (Actual/Expected); z-score: ((Actual-Expected)/sqrt(variance)); p-value: probability to have the given value of Actual or higher (or lower for negative z-score); Input IDs: original probe/gene IDs in the activated dataset(s); Signal: variation of gene expression in log_2_ FC; p-value2: statistical significance in the differential expression analysis. (XLS 52 kb)

**Additional file 8: Table S8.** Transcription factors (TFs) prediction for susceptible hosts. Network analysis predicted potential TFs underlying the regulation of gene expression, based on the variation of TopDEGs. The output file was manually-curated, and only the TFs found in cattle were classified according to their z-score. TopDEGs TFs were also indicated with their respective modulation and statistical significance. Network object name: network object name in MetaBase®; Actual: number of network objects in the activated dataset(s) which interact with the chosen object; n: number of network objects in the activated dataset(s); R: number of network objects in the complete database or background list which interact with the chosen object; N: total number of gene-based objects in the complete database or background list; Expected: mean value for hypergeometric distribution (n*R/N); Ratio: connectivity ratio (Actual/Expected); z-score: ((Actual-Expected)/sqrt(variance)); p-value: probability to have the given value of Actual or higher (or lower for negative z-score); Input IDs: original probe/gene IDs in the activated dataset(s); Signal: variation of gene expression in log_2_ FC; p-value2: statistical significance in the differential expression analysis. (XLS 48 kb)

**Additional file 9: Table S9.** Estimates of statistic parameters used to select genetically divergent hosts. Statistic estimates of the mean (mCount), mean standard deviation (mSD), minimum (minCount) and maximum (maxCount) tick counts, and estimated breeding value (EBV) are shown individually for resistant (R) and susceptible (S) hosts. (XLS 32 kb)

## Data Availability

The data supporting the conclusions of this article is available in the European Nucleotide Archive (ENA) repository (EMBL-EBI), under accession number PRJEB33196 [https://www.ebi.ac.uk/ena/browser/home].

## Abbreviations

CD: Cluster of differentiation
DC: Dendritic cell
DEG: Differentially expressed gene
EA: Enrichment analysis
EBV: Estimated breeding value
ECM: Extra-cellular matrix
FC: Fold change
FDR: False discovery rate
GO: Gene ontology
GWAS: Genome-wide association study
ID: Identification
Jak: Janus kinase
KLK: Kallikrein
LD: Linkage disequilibrium
MMP: Matrix metallopeptidase
QTL: Quantitative trait loci
R: Resistant
RNA-Seq: RNA- Sequencing
R*post*: R hosts after infestation
R*pre*: R hosts before infestation
S: Susceptible
SD: Standard deviation
SNP: Single nucleotide polymorphism
S*post*: S hosts after infestation
S*pre*: S hosts before infestation
STAT: Signal Transducers and activators of transcription
TF: Transcription factor
Th: T helper cell
TIMP: Tissue inhibitor of metalloproteinase
TopDEG(s): DEG(s) with |log_2_| FC > 1
vs.: Versus
WNT: Wingless
